# P-1177. Impact of *Staphyloccocus aureus* Surveillance and Decolonization on MRSA and MSSA Infections in a Neonatal Intensive Care Unit

**DOI:** 10.1093/ofid/ofae631.1363

**Published:** 2025-01-29

**Authors:** Nahid Hiermandi, Catherine Foster, Judith R Campbell, Krystal Purnell, Elizabeth Tocco, Lucila Marquez, Andrea Davis

**Affiliations:** Baylor College of Medicine/Texas Children's Hospital, Houston, Texas; Baylor College of Medicine, Houston, TX; Baylor College of Medicine, Houston, TX; Texas Children's Hospital, Houston, Texas; Texas Children's Hospital, Houston, Texas; Baylor College of Medicine, Houston, TX; Texas Children's Hospital, Houston, Texas

## Abstract

**Background:**

*Staphyloccus aureus* (SA) infection causes significant morbidity and mortality in NICU patients. Colonization by SA is a risk factor for infection. Surveillance and decolonization aims to curb the transmission of SA isolates thereby reducing rates of SA infection.

**Figure 1.**
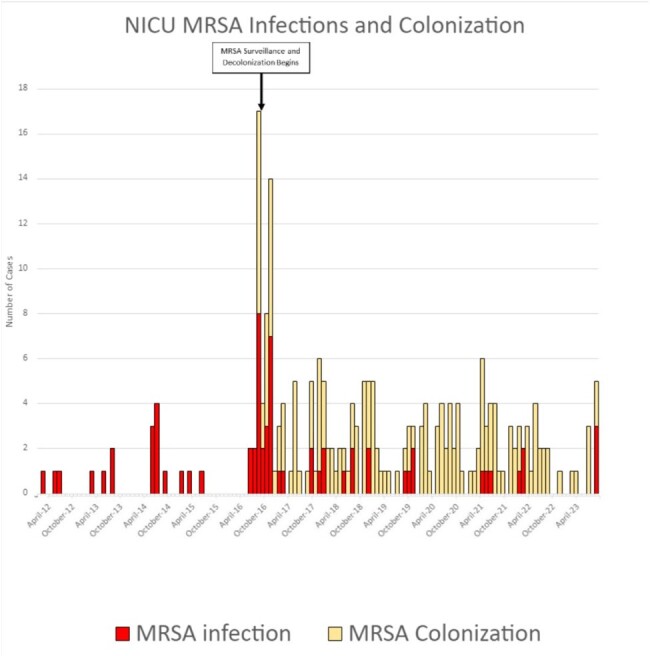
Epidemic Curve of MRSA Colonization and Infections 2012-2023

**Methods:**

This retrospective study from 2012-2023 takes place in a 42-bed level-three NICU in Texas Children’s Hospital (Houston, Texas). Neonates are admitted to a private room and undergo weekly surveillance from admission/birth until discharge. Surveillance cultures for MRSA and subsequent decolonization of MRSA-positive neonates began in Oct. 2016. MRSA surveillance was changed to PCR (with reflex to culture if positive) in June 2017. Surveillance PCR (with reflex to culture if positive) and decolonization for MSSA began in Oct. 2021. Colonization is defined as detection by PCR or isolation by culture of SA from nares, umbilicus, or groin. Colonized neonates are treated with mupirocin twice daily for five days and if at least 4 weeks chronologic age or 36 weeks gestational age, topical 2% chlorhexidine wipes daily for five days. Infection is defined by the isolation of SA from cultures in the context of illness. Multiple positive specimens from the same site are considered the same infection if isolated within a 14-day window. Epidemic curves compare colonization and infections before and after the protocol. This study was approved by the Baylor College of Medicine Institutional Review Board.

**Figure 2.**
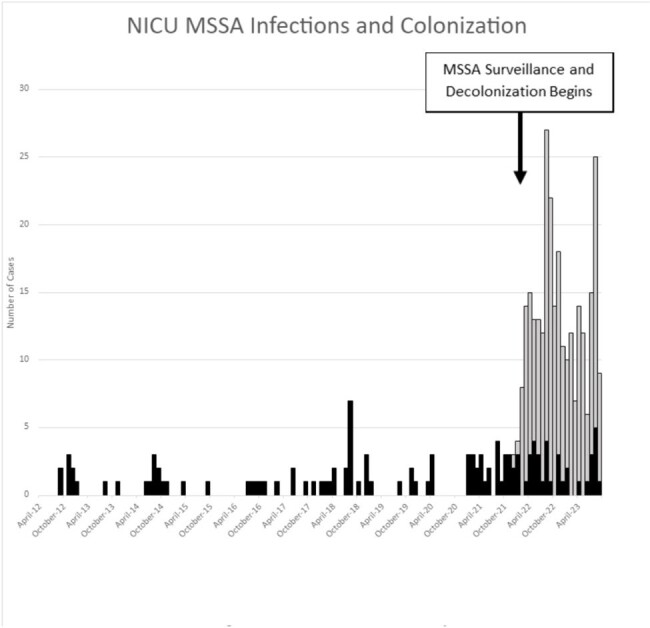
Epidemic Curve of MSSA Colonization and Infections 2012-2023

**Results:**

Surveillance and decolonization of MRSA-colonized neonates reduced the number of MRSA infections from its peak in 2016 (Figure 1). We continue to see SA-colonized infants especially with a substantial MSSA colonization burden. Despite the high rate of MSSA colonization, the number of MSSA infections remains low (Figure 2). Control charts (Figure 3 and 4) demonstrate rate, mean and upper limit of infection rates calculated per 1000 patient days.

**Figure 3.**
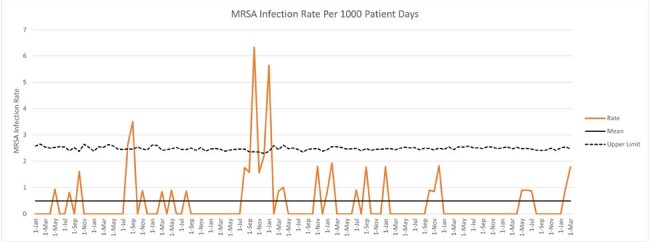
Control Chart for MRSA Infections

**Conclusion:**

The implementation of surveillance and targeted decolonization has been shown to decrease the number of MRSA infection even with ongoing colonization. Despite the high burden of MSSA-colonization, we do not see major peaks of MSSA infections. Further studies should explore if the presence of resistance genes to mupirocin and/or chlorhexidine explains ongoing colonization.

**Figure 4.**
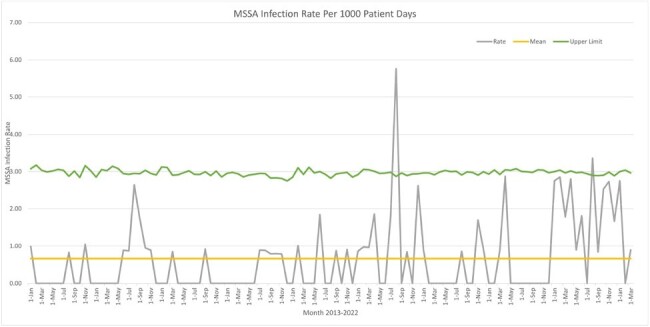
Control Chart for MSSA Infections

**Disclosures:**

**All Authors**: No reported disclosures

